# Consistent Sex-Specific Patterns in the Anatomy of the Femoral and Profunda Femoris Arteries: A Cadaveric Study

**DOI:** 10.7759/cureus.100849

**Published:** 2026-01-05

**Authors:** Slobodan Kapor, Enis Cezayirli, Predrag Bjelogrlic, Drazan Eric, Vuk Djulejic

**Affiliations:** 1 Neuroanatomy, Institute of Anatomy "Niko Miljanic" School of Medicine, University of Belgrade, Belgrade, SRB; 2 Anatomy, University of St Andrews, St Andrews, GBR; 3 Clinical Skills, University of St Andrews, St Andrews, GBR; 4 Plastic Surgery, Al Emadi Hospital, Doha, QAT; 5 Anatomy, Institute of Anatomy "Niko Miljanic“ School of Medicine, University of Belgrade, Belgrade, SRB

**Keywords:** anatomical variation, cadaveric study, femoral artery, femoral artery access, profunda femoris artery, sexual dimorphism

## Abstract

Background

The variation of the femoral artery and profunda femoris artery (FA and PFA) anatomy is clinically significant with regard to FA access and surgery of the femoral region. Despite the variability of these vessels having been extensively reported, sex-biased anatomical differences are yet to be more thoroughly investigated. In this work, we attempted to examine sex-specific morphometry, origin, and branching patterns of the FA and PFA from a morphometric, origin-to-branch morphology/strand-to-cross-sectional perspective.

Methods

This cadaveric study analyzed 96 femoral regions obtained from 48 adult cadavers, including 24 male individuals and 24 female individuals. The FA's origin from the inguinal canal was measured bilaterally. The location of origin of the PFA was evaluated with regard to the adductor longus and sartorius muscles, and the terminal branches were recorded. Statistical analysis included independent-samples t-tests, nonparametric testing as indicated, and effect size calculations.

Results

The mean distance between the origin of the FA and inguinal canal was greater in the male cadavers than in female cadavers (2.63 ± 0.38 cm vs. 2.02 ± 0.44 cm; p<0.001; Cohen’s d=1.48). There was no left-right asymmetry. Sex-specific patterns of arterial origin of PFA were found to be consistent. In female cadavers, the artery originated at the proximal one-third of adductor longus and distal half of sartorius muscle, and in male cadavers, it originated at the mid-portion of adductor longus and proximal one-third of sartorius muscle. Male cadavers had a more abundant mean number of terminal branches of the PFA (3.50 ± 0.55 vs 2.67 ± 0.52 in female cadavers), though this difference was not significant.

Conclusion

This study demonstrates pronounced and consistent sex-related anatomical differences in the FA and PFA. Recognition of these differences is essential for improving the safety and accuracy of femoral artery access and for optimizing surgical planning in orthopedic, vascular, and reconstructive procedures involving the femoral region.

## Introduction

The femoral artery (FA) is the principal arterial vessel of the lower limb [1]. It represents the direct continuation of the external iliac artery and extends from the midpoint of the inguinal ligament to the adductor hiatus (hiatus adductorius), where it continues as the popliteal artery [2]. The FA enters the thigh through the vascular lacuna (lacuna vasorum) of the subinguinal space and courses within a musculofascial canal between the anterior and medial compartments of the thigh. This canal is divided by the sartorius muscle into the femoral triangle (trigonum femorale, Scarpa’s triangle) proximally and the adductor canal (Hunter’s canal) distally [2]. The FA exists in a superficial position during its first third of thigh extension, while it runs through the femoral triangle, but moves to a deeper position in the adductor canal during the middle section of the thigh, where it receives protection from the sartorius muscle. The femoral nerve is located lateral to the FA, whereas the femoral vein lies medial to the artery in the proximal portion of the femoral triangle and gradually passes posterior to it near the apex of the triangle [2,3]. Within the adductor canal, the femoral vein is positioned posterior and lateral to the artery [2]. The saphenous nerve, a terminal branch of the femoral nerve, initially lies lateral to the femoral artery in the adductor canal, crosses anterior to it, and subsequently continues medially. The femoral triangle contains the FA which produces five essential branches. These include the superficial epigastric artery, superficial circumflex iliac artery, inguinal branches, external pudendal arteries, and deep femoral artery (profunda femoris artery or PFA). The adductor canal contains a single branch which emerges from this artery to form the descending genicular artery [3]. The FA exits the adductor canal through the adductor hiatus, located approximately four fingerbreadths proximal to the medial femoral condyle, and enters the popliteal fossa, where it continues as the popliteal artery [2]. The PFA is the largest branch of the FA and constitutes the primary blood supply to the thigh musculature, as well as contributing to the vascularization of the hip joint and femur [4,5]. In addition to numerous muscular branches, the PFA typically gives rise to the medial and lateral circumflex femoral arteries and multiple perforating arteries [2].

Medical practice is further complicated by the presence of variable anatomical patterns of the FA and its branches. The FA serves as a common entry point for medical procedures which include cardiac catheterization and coronary angiography so healthcare providers need to understand its exact path and visible markers [1]. The performance of percutaneous coronary intervention procedures results in vascular access complications which cause major bleeding events that affect 2% of patients who receive treatment. The incidence of femoral access complications requires healthcare providers to use dependable anatomical landmarks which help them perform successful arterial punctures and avoid complications [6]. With respect to FA branching patterns, variations in the origin and course of the PFA are of particular relevance during diagnostic and surgical procedures in the femoral region [7]. The knowledge of these different anatomical structures helps medical staff prevent complications which include secondary hemorrhage that can occur during FA puncture [4]. Plastic and reconstructive surgery requires complete understanding of PFA origins together with its branching patterns because this knowledge enables doctors to stop flap tissue from developing ischemia or necrosis [8].

The aim of the present study was to determine the level of origin of the FA, its distance from the inguinal ligament, and its site of termination. Additionally, we sought to analyze the level of origin of the PFA and the number of its terminal branches.

## Materials and methods

Study design

The research used descriptive cadaveric anatomical methods to study how female and male bodies differ in their FA and PFA structures and their connections and dimensions.

Study sample

The study was conducted on 48 adult human cadavers (24 male cadavers and 24 female cadavers) obtained through a legally regulated body donation program at the Department of Anatomy (Institute of Anatomy “Niko Miljanic”, Belgrade, Serbia, and School of Medicine, University of St Andrews, Scotland). A total of 96 femoral regions were analyzed, comprising 48 measurements from the female specimens and 48 from the male specimens. The research excluded cadavers that showed evidence of previous surgical procedures, traumatic injuries, congenital deformities, and gross pathological changes in the femoral region because these conditions would distort the anatomical structure.

Ethical considerations

The cadavers became available for educational and scientific research through voluntary donation after their donors gave consent before their death. The research followed institutional ethical rules which also complied with national laws that controlled the application of human anatomical specimens. No identifying information related to donors was available to the investigators.

Dissection procedure

Standard anatomical dissection of the femoral region was performed bilaterally. The cadavers placed in supine placement with their lower limbs positioned in a slightly abducted and externally rotated position to achieve best access to the femoral triangle and medial thigh area. A longitudinal skin incision was made along the anterior aspect of the thigh, extending from the inguinal ligament to the distal third of the thigh. The team performed a complete assessment of skin and subcutaneous tissue while they maintained the original condition of superficial veins and nerves. The fascia lata was then incised and reflected to expose the femoral triangle. The FA was identified inferior to the inguinal ligament within the femoral triangle, lateral to the femoral vein and medial to the femoral nerve. The artery was traced proximally to determine its point of origin relative to the inguinal canal.

Identification of anatomical structures

**Figure 1:**
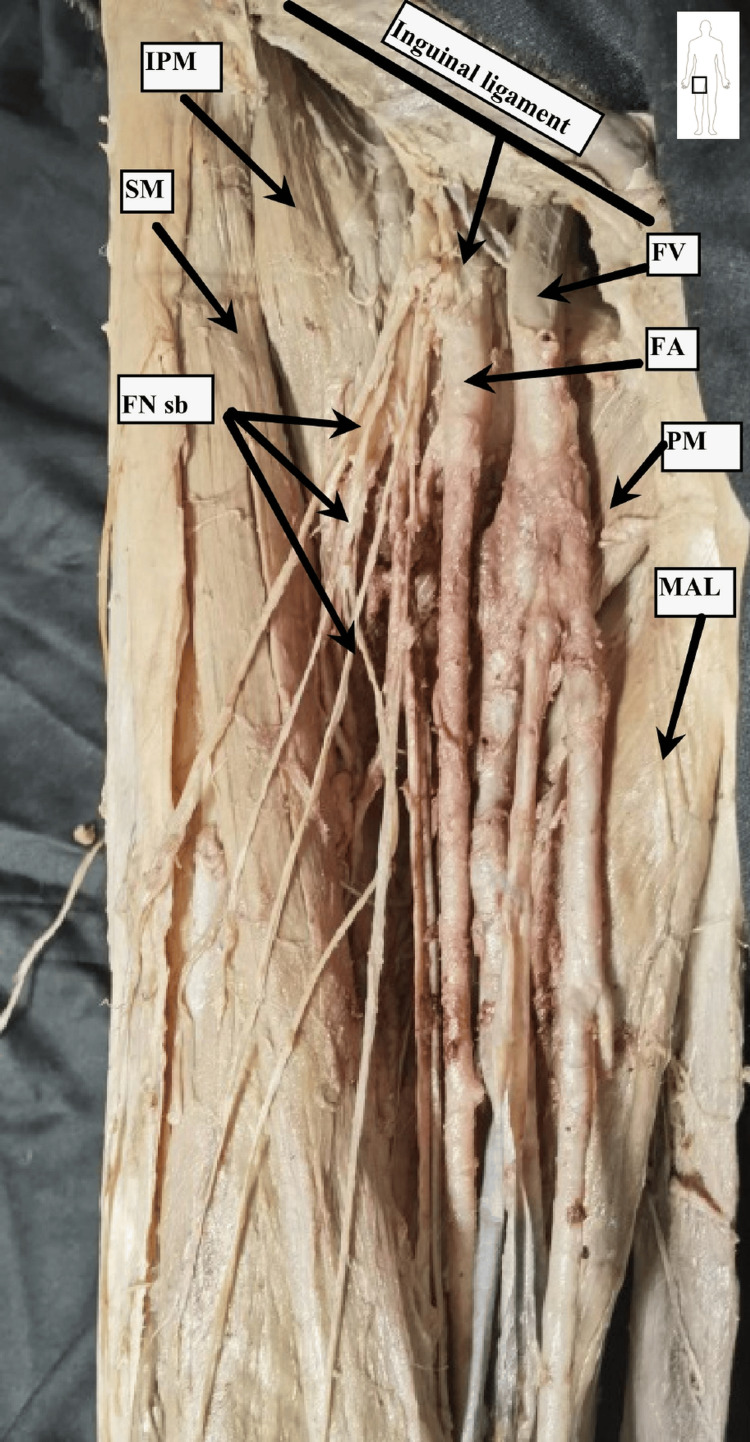
Dissection procedure of the right femoral triangle The black line represents the projection of the inguinal ligament. The arrow from the inguinal ligament shows the methodology of measuring the distance between the inguinal ligament and the femoral artery. Dissection performed by Dr. Kapor S. Marked structures are: IPM, iliopsoas muscle; SM, sartorius muscle; MAL, adductor longus muscle; PM, pectineus muscle; FV, femoral vein; FA, femoral artery; FNsb, femoral nerve sensory branches.

**Figure 2:**
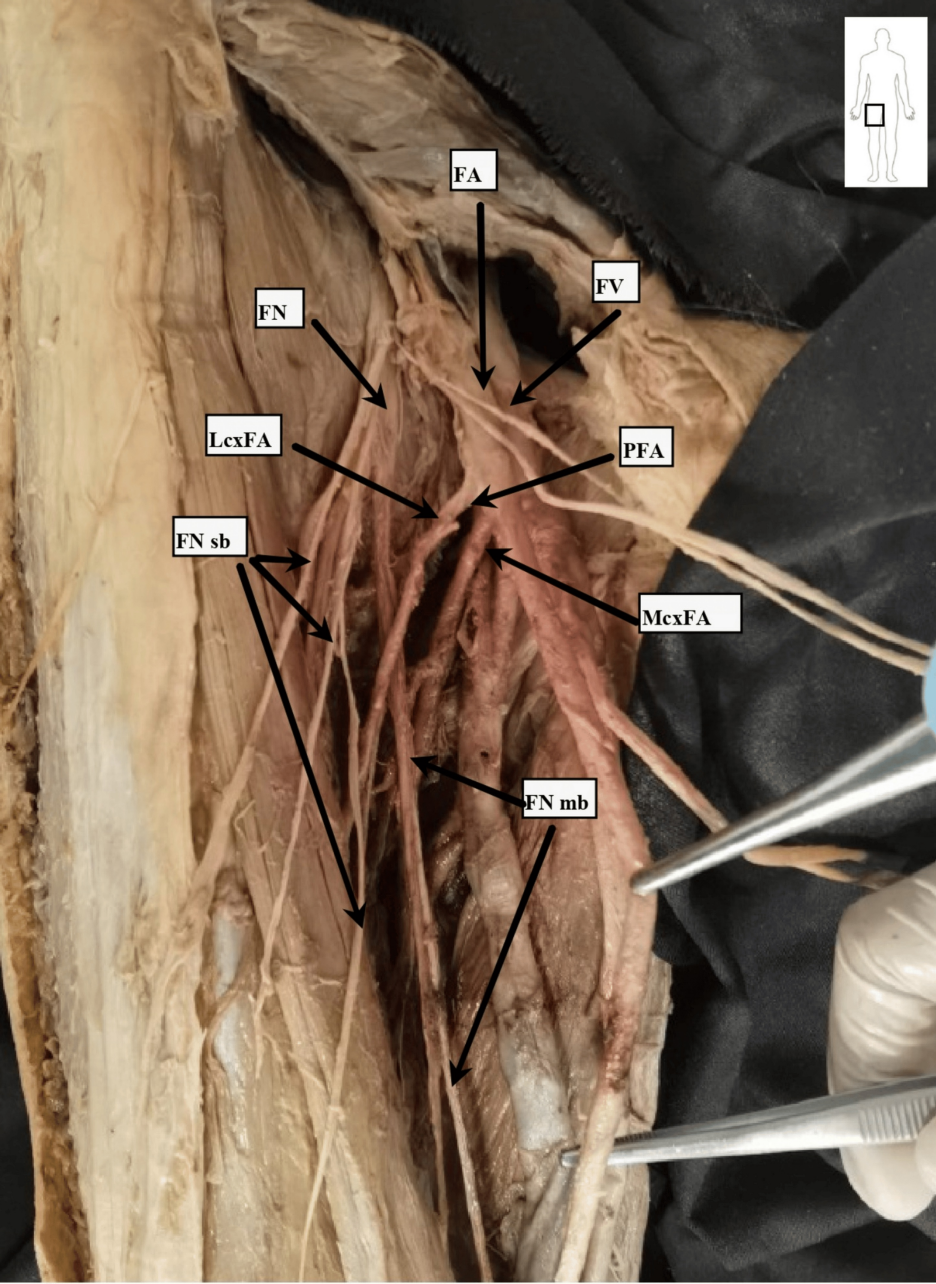
Dissection procedure of the deep structures in the right femoral triangle Dissection performed by Dr. Kapor S. Marked structures are: FA, femoral artery; FV, femoral vein; PFA, profunda femoris artery; LcxFA, lateral circumflex femoral artery; MxcFA, medial circumflex femoral artery; FN, femoral nerve; FNsb, femoral nerve sensory branches; FNmb, femoral nerve muscular branches.

**Figure 3:**
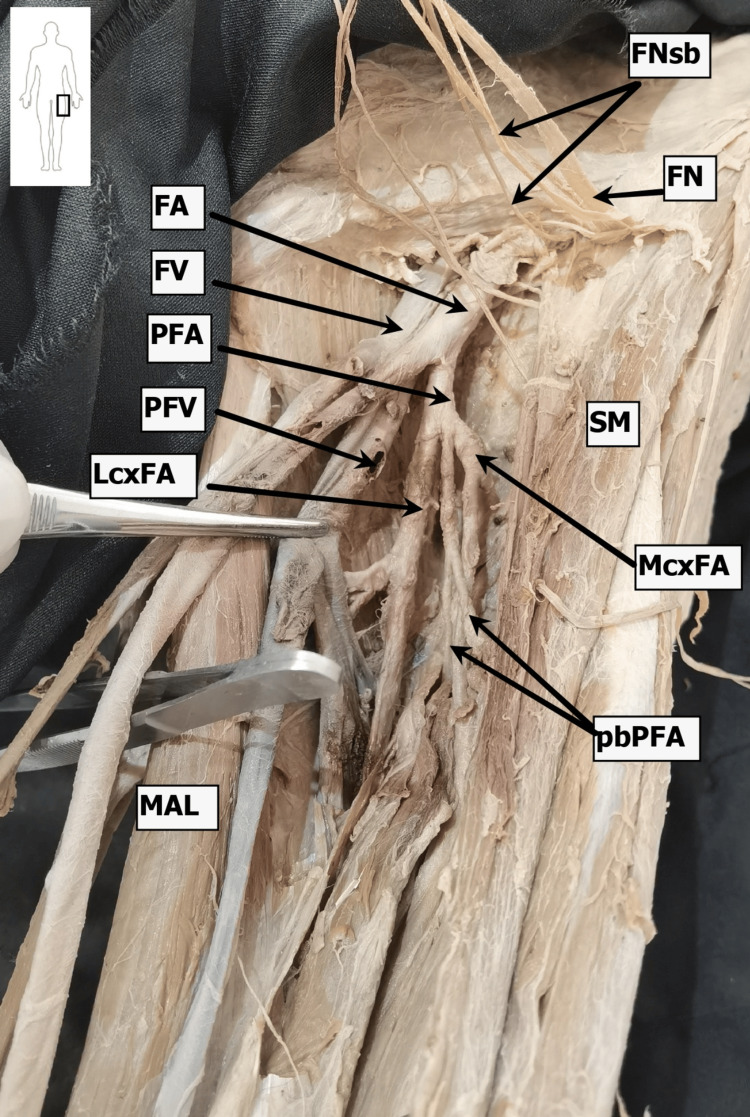
Dissection procedure of the deep structures in the left femoral triangle Dissection performed by Dr. Kapor S. Marked structures are: FA, femoral artery; FV, femoral vein; PFA, profunda femoris artery; PFV, profunda femoris vein; LcxFA, lateral circumflex femoral artery; MxcFA, medial circumflex femoral artery; FN, femoral nerve; FNsb, femoral nerve sensory branches; MAL, adductor longus muscle; SM, sartorius muscle; pbFA, perforating (terminal) branches of the femoral artery.

During dissection, key anatomical landmarks and vascular structures of the femoral region were identified and documented (Figures 1-3).

The FA was identified as the continuation of the external iliac artery distal to the inguinal ligament and served as the primary reference structure. The inguinal canal was used as a fixed anatomical landmark for measuring the distance to the FA origin. The PFA was identified as the largest posterior branch arising from the FA and was traced distally. Its relationship to the adductor longus muscle was assessed to determine whether its origin corresponded to the proximal one-third or the mid-portion of the muscle. The sartorius muscle was identified along its oblique course across the femoral triangle and used as an additional reference for classifying the origin of the PFA as corresponding to either its proximal one-third or distal half. Finally, the PFA was followed distally to identify and count its terminal branches extending into the posterior compartment of the thigh.

Morphometric measurements

The distance between the origin of the FA and the inguinal canal was measured using a digital caliper with millimeter precision. The measurements followed the direction which ran perpendicular to the inguinal canal and the researchers documented them in centimeter units. The measurements took place on both sides of the body while the researcher worked independently to achieve precise results. The site of origin of the PFA was classified according to its vertical position relative to the adductor longus and sartorius muscles. The research team documented all observations by taking photos and used standardized data collection sheets to document the recorded information.

Statistical analysis

The research presented mean values with standard deviation (SD) for continuous variables. Side-related differences in the FA origin distance were analyzed using a paired Student’s t-test. The independent-samples t-test with Welch correction performed an analysis to determine if sex-related differences existed between groups. Categorical anatomical variables were analyzed descriptively and presented as frequencies and percentages. Differences in the number of terminal branches of the PFA were evaluated using the Mann-Whitney U test. The researchers used effect size calculations to determine the size of the measured differences between groups. The researchers established a p-value of less than 0.05 as their threshold for statistical significance. The research team conducted their statistical analysis through IBM SPSS Statistics for Windows, Version 11 (Released 2002; IBM Corp., Armonk, New York, United States) which they used as their standard statistical tool.

## Results

A total of 96 femoral regions were analyzed, including 48 measurements obtained from the female specimens and 48 from the male specimens. The analyzed parameters included the distance between the origin of the FA and the inguinal canal, the site of origin of the PFA in relation to surrounding musculature, and the number of terminal branches of the PFA. All measurements were analyzed with respect to sex, and bilateral symmetry was assessed where applicable.

Distance of the FA origin in relation to the inguinal canal

The distance between the origin of the FA and the inguinal canal ranged from 1.5 to 3.0 cm in female specimens and from 2.0 to 3.0 cm in male specimens. The mean FA-inguinal canal distance was 2.02 ± 0.44 cm in the former and 2.63 ± 0.38 cm in the latter. Statistical analysis using an independent-samples t-test with Welch correction demonstrated a highly statistically significant difference between sexes, with male specimens exhibiting a greater FA-inguinal canal distance compared to the female specimens (p<0.001). The magnitude of this difference was large, as indicated by a Cohen’s d value of 1.48. All these results are presented in Table 1.

**Table 1:** Distance of the femoral artery (FA) origin in relation to the inguinal canal *statistically significant.

Sex	n	Mean ± SD (cm)	Range (cm)	Statistical test	p-value	Effect size
Females	48	2.02 ± 0.44	1.5–3.0	Welch t-test	<0.001	d=1.48
Males	48	2.63 ± 0.38*	2.0–3.0			

Site of origin of the PFA in relation to the adductor longus muscle

In all the female specimens (100%), the PFA originated at the level of the proximal one-third of the adductor longus muscle. In contrast, in all male specimens (100%), the artery originated at the level of the mid-portion (one-half) of the adductor longus muscle. No left-right differences were observed in either sex, indicating complete bilateral symmetry in the site of origin of the PFA relative to the adductor longus muscle. All these results are presented in the Table 2.

**Table 2:** Site of origin of the profunda femoris artery (PFA) in relation to the adductor longus muscle (MAL)

Sex	Proximal 1/3 of MAL	Mid-portion (1/2) of MAL	Bilateral symmetry
Females (n = 48)	100%	0%	Present
Males (n = 48)	0%	100%	Present

Site of origin of the PFA in relation to the sartorius muscle

With respect to the sartorius muscle, the origin of the PFA demonstrated a consistent sex-specific pattern. In female specimens (n=48), the artery originated at the level of the distal half of the sartorius muscle in all cases (100%). In male specimens (n=48), the origin corresponded to the proximal one-third of the sartorius muscle in all cases (100%). As with the relationship to the adductor longus muscle, no bilateral asymmetry was detected in either sex.

Number of the terminal branches of the PFA

The number of terminal branches of the PFA ranged from two to three in the female specimens (n=48) and from three to four in the male specimens (n=48). The mean number of terminal branches was 2.67 ± 0.52 in the former and 3.50 ± 0.55 in the latter. Comparison between sexes using the Mann-Whitney U test did not reveal a statistically significant difference (p=0.065). However, the calculated effect size was large (r=0.52), indicating a notable trend toward a higher number of terminal branches in male specimens.

Sex-related differences were identified in multiple anatomical parameters. The male specimens demonstrated a significantly greater distance between the FA origin and the inguinal canal. The site of origin of the PFA showed complete sex-specific distribution in relation to both the adductor longus and sartorius muscles, with no side-related variation. Additionally, the male specimens exhibited a higher mean number of terminal branches of the PFA, although this difference did not reach statistical significance. All key findings are presented in Table 3.

**Table 3:** Summary of key findings FA, femoral artery; PFA, profunda femoris artery; SM, sartorius muscle; MAL, adductor longus muscle.

Parameter	Females	Males	Main finding
FA-IC distance	2.02 ± 0.44 cm	2.63 ± 0.38 cm	Significantly greater in male specimens (p<0.001)
PFA origin vs. MAL	Proximal 1/3 (100%)	Mid-portion (100%)	Complete sex-related difference
PFA origin vs. SM	Distal 1/2 (100%)	Proximal 1/3 (100%)	Complete sex-related difference
PFA terminal branches	2–3	3–4	Higher number in males (trend)
Bilateral symmetry	Present	Present	No side-related variation

## Discussion

The current cadaveric analysis highlights a detailed study of sex- and morphology-related anatomical differences between the FA and PFA, including morphometric measurements and the anatomical relationship of their origins to the surrounding muscles, along with the terminal branching arrangements for the functional anatomy of the FA and PFA among male and female specimens. The findings revealed consistent sexual dimorphism, which was incompletely described in the previous anatomical findings. Among the significant findings was the statistically significant difference in the distance between the root of the FA and the inguinal canal, which in the male specimens was significantly higher than in the female specimens. Most previous cadaveric and imaging investigations have concentrated on the variability of the FA and PFA with respect to the mid-inguinal point or inguinal ligament and have indicated variation in the degree of origin [7,9-11]. However, the majority of these studies failed to conduct a sex-directed comparison. Nasr et al. reported that the distance ranges among male and female specimens overlapped but did not show significant sex differences [7]. Conversely, the current finding suggests a highly significant difference with a very large effect size, and thus suggests sexual dimorphism in the morphology of the FA may be more pronounced than previously reported.

The lack of left-right asymmetry of the origin of the FA appearing in our study agreed with most prior studies, which revealed no significant lateral variation in the anatomy of FA or PFA [10,12]. This bilateral symmetry is indicative of the validity of anatomical landmarks commonly used for FA access and suggests that sex-related differences may be more clinically relevant sources of variability. Among the few newly available findings is a sex-specific PFA origin structure in accordance with the adductor longus and sartorius muscles. Although a number of studies have reported the origin of the PFA as an anterior (posterolateral), posterior, lateral, or medial feature of the FA wall [4,7,9-12], fewer have linked its origin with muscular landmarks in a sex-stratified fashion. The uniformity of the expression of PFA localization at the proximal one-third of the adductor longus and distal half of the sartorius in women and the mid-portion of the adductor longus and proximal one-third of the sartorius in men is a peculiar, hitherto unknown anatomic characteristic. This result contributes to the classification of where the PFA origin can be derived from and is a guide for surgical and intra-operative treatment on the femoral plane. The obvious bilateral pure symmetry of the PFA origin of the subjects differs from some other reports which reported occasional asymmetry, or rare cases of PFA like double PFAs or anomalous origins of PFA origin from the external iliac artery [13,14]. The lack of these variants in the current series might be indicative of population variability or due to the small sample size. However, they were internally consistent and confirmed their anatomical accuracy.

In terms of branching in the PFA at terminal point, male specimens were more likely to have more terminal branches. While this difference was not statistically significant, the high effect size suggests a significant anatomical pattern. In previously described cadaveric studies, varying numbers of perforating branches derived from PFA have been documented, typically between two to four [7,9,11]. Even so, these types of studies did not consider sex differences in branching designs. The pattern evident in our study indicates that the vascular complexity may be practically relevant in the case of surgical dissection or reconstruction in the posterior thigh compartment in male individuals. Therefore, these results are in concordance with other literature on the variability of the FA and PFA, and add evidence for consistent, clinically applicable sex-related variation across the different anatomical areas. These findings stress the need to include sex-specific anatomical parameters in anatomical education and also in clinical practice at all times, especially those related to procedures involving access to the FA, orthopedic surgery, and reconstructive flap retrieval. The research results from this study provide essential clinical information which benefits medical staff who perform FA procedures and surgeons who operate in the femoral area.

The larger space between the FA origin and inguinal canal in male subjects requires healthcare providers to use sex-specific methods when performing FA cannulation, cardiac catheterization, and endovascular procedures because wrong puncture locations can lead to vascular complications, including hemorrhage, pseudoaneurysm development, and arteriovenous fistula formation [1,6]. The shorter distance which female subjects exhibit might help explain why access-related complications occur more frequently in them according to specific clinical studies, and also demonstrates the need for exact anatomical landmark identification when treating female patients [6]. The PFA demonstrates sex-specific patterns which surgeons must understand when performing orthopedic and vascular and reconstructive procedures in the femoral area. The PFA origin shows different positions, which surgeons need to identify because failure to do so can cause blood vessel damage during operations or needle procedures that produce dangerous bleeding [4,7]. Plastic and reconstructive surgery requires a complete understanding of the PFA anatomy to perform anterolateral thigh flap harvesting successfully [5].The observed pattern, which showed male patients having more terminal branches of the PFA than female patients, did not reach statistical significance, but it could make surgical procedures more complicated and dangerous for blood vessel damage in the posterior thigh area. Doctors can apply their knowledge of blood vessel patterns to create better preoperative plans which results in safer surgical methods and decreased surgical problems [5,8].

The present study has several limitations that should be acknowledged. The relatively small sample size may limit the generalizability of the findings and reduce the statistical power for certain comparisons, particularly regarding the number of terminal branches of the PFA. In addition, the cadaveric nature of the study does not account for physiological factors present in living individuals, such as vascular tone or positional changes. Finally, potential population-specific anatomical characteristics cannot be excluded. Despite these limitations, the consistent sex-related patterns observed in this study provide valuable anatomical and clinically relevant insights.

## Conclusions

This cadaver investigation demonstrates distinct and stable sex differences in the morphology of the FA and PFA. Male subjects show a larger gap between the FA origin and the inguinal canal compared to female subjects, which implies more pronounced sexual dimorphism in the femoral vascular anatomy. Also, the site of origin of the PFA follows a uniform, sex-specific pattern, symmetrical to both sides, in relation to the adductor longus and sartorius muscles. Male subjects also demonstrated a higher number of terminal branches in the PFA, indicating more vascular complexity. These findings add to prior anatomical understanding and highlight the need for incorporating sex-specific anatomical considerations into clinical practice. Knowledge and understanding of these differences could, therefore, aid in developing efficient and safe access to the FA, minimize the likelihood of vascular complications, and facilitate accurate surgical planning during orthopedic, vascular, and reconstructive procedures, including those of the femoral region.
